# Absence of the TAP2 Human Recombination Hotspot in Chimpanzees

**DOI:** 10.1371/journal.pbio.0020155

**Published:** 2004-06-15

**Authors:** Susan E Ptak, Amy D Roeder, Matthew Stephens, Yoav Gilad, Svante Pääbo, Molly Przeworski

**Affiliations:** **1**Max Planck Institute for Evolutionary AnthropologyLeipzigGermany; **2**Department of Statistics, University of WashingtonSeattle, WashingtonUnited States of America

## Abstract

Recent experiments using sperm typing have demonstrated that, in several regions of the human genome, recombination does not occur uniformly but instead is concentrated in “hotspots” of 1–2 kb. Moreover, the crossover asymmetry observed in a subset of these has led to the suggestion that hotspots may be short-lived on an evolutionary time scale. To test this possibility, we focused on a region known to contain a recombination hotspot in humans, TAP2, and asked whether chimpanzees, the closest living evolutionary relatives of humans, harbor a hotspot in a similar location. Specifically, we used a new statistical approach to estimate recombination rate variation from patterns of linkage disequilibrium in a sample of 24 western chimpanzees *(Pan troglodytes verus).* This method has been shown to produce reliable results on simulated data and on human data from the TAP2 region. Strikingly, however, it finds very little support for recombination rate variation at TAP2 in the western chimpanzee data. Moreover, simulations suggest that there should be stronger support if there were a hotspot similar to the one characterized in humans. Thus, it appears that the human TAP2 recombination hotspot is not shared by western chimpanzees. These findings demonstrate that fine-scale recombination rates can change between very closely related species and raise the possibility that rates differ among human populations, with important implications for linkage-disequilibrium based association studies.

## Abstract 2

The human TAP2 recombination hotspot is absent from the homologous region in western chimpanzees, with important implications for association studies, the HapMap project and understanding fine-scale variation in recombination rates

## Introduction

Recombination is a fundamental biological feature about which we still know remarkably little, especially in mammals. Understanding recombination is also of practical importance for evolutionary inference and human genetics (Nachman 2002; Arnheim et al. 2003). Unfortunately, the process is difficult to study, because recombination events occur extremely rarely (e.g., with a probability of ∼10^−8^ per bp per generation in a typical region of the human or Drosophila melanogaster genome; Ashburner 1989; Kong et al. 2002). Thus, direct measurements for closely linked sites often require the examination of a prohibitive number of individuals. As a result, our knowledge of recombination rates stems primarily from estimates for markers that are megabases apart, obtained from crosses or, for humans, obtained from pedigrees (e.g., Kong et al. 2002).

One way to learn about finer-scale recombination rates in males is sperm typing (Li et al. 1988; Hubert et al. 1994; Jeffreys et al. 2001). In this approach (reviewed by Arnheim et al. 2003), genetic markers are amplified and typed from a large number of sperm in order to estimate the fraction of recombinant sperm and hence the recombination rate. Fine-scale rates can also be measured indirectly from patterns of allelic associations, or linkage disequilibrium (LD), observed in samples from natural populations (Hudson 1987; Pritchard and Przeworski 2001). In humans, both direct estimates of the recombination rate using sperm typing techniques and indirect approaches based on analyses of LD have suggested the existence of substantial heterogeneity in rates of recombination at small scales (Daly et al. 2001; Jeffreys et al. 2001; Gabriel et al. 2002; Schneider et al. 2002; Wall and Pritchard 2003). In particular, sperm typing experiments have demonstrated that, in several regions of the human genome, crossover resolutions are not uniformly distributed but instead tend to cluster within narrow regions of 1–2 kb termed “recombination hotspots” (de Massy 2003 and references therein).

While there has been recent progress characterizing the extent of spatial variation in recombination rates, the time scale over which recombination rates change remains an open question. It has been known for decades that natural populations harbor genetic variation for recombination rates (Brooks 1988 and references therein). In humans, in particular, there are significant differences in recombination rates among females (Kong et al. 2002) as well as among males (Cullen et al. 2002). Thus, there is a clear potential for the evolution of recombination rates. However, there are only a couple of demonstrated cases that help to delimit the time scale on which this might occur: at the megabase scale, the best example is probably D. melanogaster and *D. simulans,* two sibling species that differ in their recombination landscape (True et al. 1996). Among primates, the genetic map of humans is approximately 28% longer than that of an Old World monkey, the baboon (*Papio hamadryas;* Rogers et al. 2000), suggesting that—if physical maps are roughly similar—recombination rates in humans may be higher overall. These instances demonstrate that large-scale recombination rates can change between species that differ on average at roughly 6% to 10% of nucleotide positions (Betancourt and Presgraves 2002; Thomas et al. 2003).

At a finer scale, the only evidence stems from a recent study of the *β-globin* gene, where a hotspot had been characterized by sperm typing in humans. Wall et al. (2003) found no evidence of rate variation in LD data collected from the rhesus macaque *(Macaca mulatta),* another Old World monkey. For more closely related species, nothing is known. However, observations in yeast (e.g., Petes 2001; Steiner et al. 2002) and mammals (Jeffreys and Neumann 2002; Yauk et al. 2003) raise the possibility that local recombination rates could change rapidly. Indeed, at the MS32 and DNA2 hotspots in humans (Jeffreys et al. 1998; Jeffreys and Neumann 2002) as well as at the E*_β_* hotspot in mice (*Mus* sp.; Yauk et al. 2003), some haplotypes were found to lead to higher rates of initiation of crossover events. Such haplotypes tended to be undertransmitted in crossover products (Jeffreys and Neumann 2002), an asymmetry that favors the loss of recombination hotspots (Boulton et al. 1997). If this is a common phenomenon, it may lead hotspots to be short-lived on an evolutionary time scale (Jeffreys and Neumann 2002).

To evaluate whether fine-scale recombination rates can change rapidly, we were interested in comparing rates in humans with those in their closest evolutionary relative, the chimpanzee *(Pan troglodytes).* The two species are thought to have had a common ancestor five to six million years ago and differ at approximately 1.2% of base pairs on average (Ebersberger et al. 2002). Since it is difficult to use sperm typing techniques in chimpanzees, not least of all because of the need for chimpanzee sperm, we took an indirect approach and estimated the extent of recombination rate variation from patterns of LD in a population sample. To do so, we modified a recently developed statistical approach (Li and Stephens 2003). The method estimates recombination rates by exploiting the fact that patterns of LD reflect the rate and distribution of recombination events in the ancestors of the sample (see Materials and Methods for more details). Although it is based on simplistic assumptions about population demography, it has been shown to produce reliable estimates of recombination rates for data sets simulated under a range of demographic assumptions (Li and Stephens 2003; D. C. Crawford, T. Bhangale, N. Li, G. Hellenthal, M. J. Rieder, et al., unpublished data). We focused on the TAP2 genic region, where a sperm typing study of humans characterized a ∼1.2 kb recombination hotspot in one of the introns (Jeffreys et al. 2000). Application of the statistical method to polymorphism data collected for this region (Jeffreys et al. 2000) led to estimates similar to those obtained by sperm typing, providing further evidence for its reliability (Li and Stephens 2003).

Samples that include individuals from diverged populations are expected to harbor high levels of LD that may lead to incorrect estimates of recombination rate variation (Pritchard and Przeworski 2001). This is of particular concern in chimpanzees, for which previous studies have reported high levels of genetic differentiation between subspecies (Morin et al. 1994; Stone et al. 2002; Fischer et al. 2004). In addition, there appears to be a high proportion of less informative, rare alleles in samples from central *(P. t. troglodytes)* but not western *(P. t. verus)* chimpanzees (Gilad et al. 2003; Fischer et al. 2004). We therefore collected polymorphism data from a sample of 24 chimpanzees that were all known to be from the western subspecies. Strikingly, we found no evidence for recombination rate variation at TAP2 in these data.

## Results

In humans, LD data for the TAP2 region were previously collected by Jeffreys et al. (2000), who resequenced ∼9.7 kb in a sample of eight individuals from the United Kingdom (UK) and found 46 single nucleotide polymorphisms (SNPs), excluding insertion-deletions. The SNPs were then typed in a sample of 30 individuals from the UK, in whom haplotypes were determined experimentally (by allele-specific PCR). We collected genotype data for the same region in western chimpanzees by resequencing 24 individuals (see Materials and Methods for details). This led to the discovery of 57 SNPs. When differences in study design are taken into account, diversity levels in western chimpanzees are very similar to those observed in samples of humans from the UK (*θ*_W_ = 0.145% versus *θ*_W_ = 0.144% per bp, respectively), consistent with previous findings (e.g., Gilad et al. 2003; Fischer et al. 2004).

**Figure 1:**
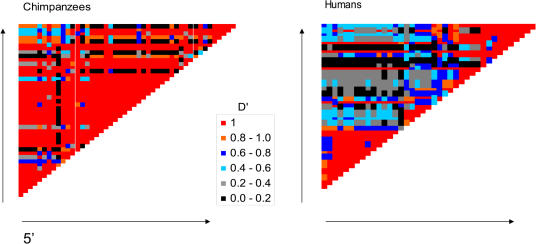
Patterns of Pairwise LD in Humans and Chimpanzees Only SNPs with minor allele frequencies above 0.1 are included. The rows correspond to the consecutive SNPs in the region, as do the columns. Each cell indicates the extent of LD between a pair of sites, as measured by |*D*′| (estimated using the Expectation Maximization algorithm, as implemented by Arlequin: http://lgb.unige.ch/arlequin/).

The LD data are summarized in Figure 1; overall, there is much less LD in humans than in chimpanzees. In particular, in humans, strong allelic associations are only seen between pairs of sites in close physical proximity, while in chimpanzees, such associations are also found among more distant pairs. Whether this reflects differences in the underlying recombination landscape or chance variation is unclear from visual inspection of these plots alone. We therefore used a statistical approach to assess the evidence for recombination rate variation in the two species. Specifically, we assumed that there is (at most) one hotspot in the region and, as a first step, specified its location according to the results of the sperm typing study in humans. We then applied our modification of the method of Li and Stephens (2003) to estimate a background population recombination rate, *ρ,* and the relative intensity of recombination in the hotspot segment, *λ* (see Materials and Methods). Within this model, a *λ* value of 1 corresponds to an absence of recombination rate variation, while values of *λ* greater than 1 indicate a hotspot. The approach taken here is Bayesian (see Materials and Methods) so, as a measure of support for a hotspot in the LD data, we report estimates for the probabilities Pr(*λ* > 1) and Pr(*λ* > 10); these are the posterior probabilities of a hotspot of any kind and of a hotspot of intensity at least ten times the background rate, respectively.

Application of this method to the human haplotype data led to extremely strong support for rate variation: we estimated Pr(*λ* > 1) = 1 and Pr(*λ* > 10) = 0.982. When the same method was applied to the human genotype data (i.e., ignoring the information about the phase of multiple heterozygotes), we estimated Pr(*λ* > 1) = 1 and Pr(*λ* > 10) = 0.992. The results are almost identical, suggesting minimal loss of information with the use of genotypes. Interestingly, the point estimate of *λ* using either haplotypes (28.4) or genotypes (32.1) is higher than the corresponding estimate from sperm typing (11). This difference may reflect error in the estimates; alternatively, it may point to a more intense hotspot in females than in males (Jeffreys et al. 2000).

**Figure 2:**
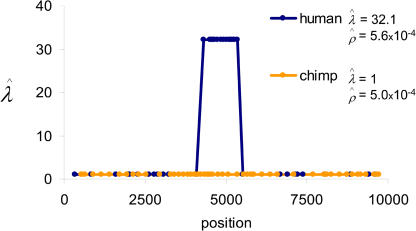
Estimates of the Recombination Hotspot Intensity, *λ,* Based on Genotype Data We assumed that, if the hotspot is present, it is in the same location as estimated by sperm typing in humans (see Materials and Methods). A *λ* value of one corresponds to the absence of recombination rate variation, while values of *λ* greater than one indicate a hotspot. The estimates for humans from the UK are shown in blue and those for western chimpanzees in orange.

Next, we applied the same method to the genotype data collected from western chimpanzees. The estimate of the background rate of recombination, ρ^*,* is 5.0 × 10^−4^ per base pair, which is very similar to the estimate from the human genotype data (Figure 2). However, in contrast to what is found in humans, there is no evidence for recombination rate variation: our estimate of *λ* is 1, suggesting a uniform rate of recombination throughout the region, and our estimates of Pr(*λ* > 1) = 0.200 and Pr(*λ* > 10) = 0.006, reflecting tepid support for a hotspot of any kind and almost no support for a hotspot similar to the one observed in humans. Indeed, the latter figure represents very strong evidence *against* a hotspot of moderate intensity and rules out the possibility that the chimpanzee polymorphism data are simply uninformative, because of, for example, insufficient sample size or diversity.

To assess how likely we would be to obtain such weak support if there were in fact a hotspot in western chimpanzees similar to the one in humans, we generated 200 simulated genotype data sets under a model with a hotspot of intensity *λ* = 11 and then tabulated the proportion with posterior probability estimates as low or lower than that observed (see Materials and Methods). We took the *λ* value estimated from sperm typing because it is the lowest of the various estimates for humans and hence its use was conservative for our purposes. With the *ρ* value estimated from the data (5.0 × 10^−4^ per bp), the probability of obtaining Pr(*λ* > 1) ≤ 0.200 is *p* = 0.010 and the probability of obtaining Pr(*λ* > 10) ≤ 0.006 is *p* = 0.005. With a lower *ρ* value (2.7 × 10^−4^ per bp; see Materials and Methods), the probability of obtaining Pr(*λ* > 1) ≤ 0.200 is *p* = 0.020. In other words, we can reject the null hypothesis that there is a hotspot in western chimpanzees similar to the one in humans, because we would expect to see more support for a hotspot in these data if one were there. It appears that western chimpanzees do not harbor a hotspot in the same location as humans.

**Figure 3:**
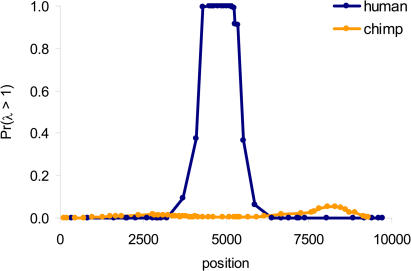
Estimates of Recombination Rate Variation in Humans and Western Chimpanzees In this model, there is at most one hotspot in the region, the location and width of which are unknown and estimated along with *λ* and *ρ.* On the *y*-axis is an estimate of the posterior probability of elevated recombination, Pr(*λ* > 1), between each pair of consecutive SNPs (plotted at the midpoint position).

The possibility remains, however, that there is a hotspot in a slightly different position in chimpanzees. To evaluate this, we used a more general model in which there is at most one hotspot in the region, but where the location is unknown and estimated together with *ρ* and *λ* (see Materials and Methods). Again, we found very little evidence for recombination rate variation: across all pairs of consecutive segregating sites, the largest posterior probability of elevated recombination is estimated to be < 0.060 (Figure 3). Thus, the hotspot appears to be entirely absent from the ∼9.4 kb surveyed in western chimpanzees.

## Discussion

These estimates of recombination rate parameters are based on assumptions of neutrality, constant population size, and random mating, raising the concern that the hotspot is not absent but instead masked by departures from model assumptions. However, we chose to focus on western chimpanzees precisely because previous studies reported allele frequencies in rough accordance with the assumptions of our model. Consistent with these studies (Gilad et al. 2003; Fischer et al. 2004), the allele frequencies at TAP2 are not significantly different from the expectations of the standard neutral model (as assessed by Tajima's *D* = 0.848, *p* = 0.237; see Materials and Methods). Moreover, simulations suggest that the power to detect a hotspot is not strongly affected by population history (Li and Stephens 2003). To some extent, this is expected, as population history tends to affect LD in the entire region, not only in the hotspot, so that estimates of the relative rates of recombination are unlikely to be substantially altered. In summary, there is no evidence for a marked departure from model assumptions in the allele frequencies, and the method is expected to be robust to small departures. Consistent with this, in humans, the approach yields similar results to sperm typing experiments that do not rely on the same assumptions. On this basis, it seems that the hotspot is truly absent from the homologous region in western chimpanzees.

This finding implies that the hotspot was lost in chimpanzees or gained in humans, or that it moved in one of the species (over a larger distance than we surveyed). This in turn raises a number of more general questions. Are hotspots frequently born de novo or do they tend to migrate within circumscribed regions of the genome? Are particular sequence motifs sufficient to produce recombination hotspots, or are larger-scale requirements, such as chromatin accessibility, required for their formation (Petes 2001)? The systematic comparison between closely related species with different recombination landscapes may be helpful in addressing these problems. As an illustration, in these data, we found two motifs that were previously implicated in the formation of recombination hotspots (Smith et al. 1998; Badge et al. 2000 and references therein) and that varied between the two species: a Pur binding motif that is present in humans but absent in chimpanzees (because of a single base pair difference) and two scaffold attachment sites that are in different positions in the two species. The significance of these differences cannot be determined on the basis of a single example; however, once a larger sample of hotspot regions has been surveyed, one can begin to test for an association between particular sequence motifs or features and the presence of hotspots.

**Figure 4:**
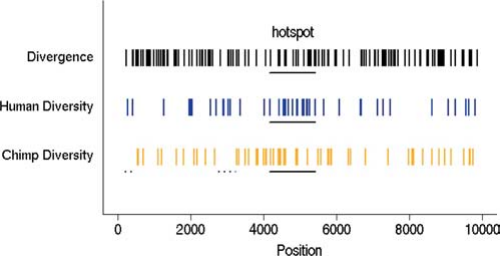
Distribution of Variable Sites in the Genomic Region The positions of sites that differ between humans and chimpanzees are shown on the first line, while the positions of sites polymorphic in humans from the UK or in western chimpanzees are shown on the next two lines. The human hotspot region is underlined. The dashed lines indicate regions not surveyed for variation in western chimpanzees (see Materials and Methods).

Comparative studies of hotspot regions will also increase our understanding of the determinants of mutation rates. As noted by Jeffreys et al. (2000), there is a significant excess of diversity within the hotspot region in humans from the UK (Figure 4): when the hotspot region is compared to the 8,735 other windows of the same size, only 0.3% have as many or more SNPs. In contrast, in western chimpanzees, levels of diversity are not higher than elsewhere in the region (Figure 4): 17.0% of comparable windows harbor at least as many SNPs as the hotspot. Nor are levels of human–chimpanzee divergence unusual in the hotspot region: 67.3% of windows show the same or higher numbers of fixed differences between species (Figure 4). Given the evidence for a recombination hotspot in humans but not in chimpanzees, these observations are consistent with an association between recombination and mutation in primates (Hellmann et al. 2003) and, in particular, with a mutagenic effect of recombination (Rattray et al. 2002). If indeed recombination events introduce mutations, the lack of a peak of human–chimpanzee divergence in the hotspot region (Jeffreys et al. 2000; Figure 4) would suggest that the hotspot arose fairly recently in human evolution.

In conclusion, these analyses demonstrate that fine-scale recombination rates can change between closely related species. Together with the observations that crossover frequencies can depend on specific haplotypes (Jeffreys and Neumann 2002) and that large-scale recombination rates differ among individuals (Cullen et al. 2002; Kong et al. 2002), this finding raises the possibility that local rates can vary among human groups that differ in their allele frequencies. Unfortunately, demonstrating compelling evidence for variation among human populations on the basis of LD data alone promises to be substantially harder than demonstrating such differences between chimpanzees and humans. In particular, human populations share most of their evolutionary history, making differences between extant populations, if they exist, more difficult to detect. Nevertheless, LD studies should be helpful in identifying interesting regions for further study via sperm typing.

The extent to which local recombination rates vary among human populations influences the degree of similarity of LD patterns among them, with important consequences for the design of efficient LD-based association studies (including, for example, the choice of appropriate “haplotype tagging SNPs” [Johnson et al. 2001] in different human populations) and for the relevance of data generated by the current human HapMap project to populations not currently represented in that study (International Hapmap Consortium 2003). Perhaps most importantly, if local recombination rates do vary among groups, then the study of regions with the most pronounced differences should lead to further insights into the underlying biological processes that cause fine-scale variation in recombination rates.

## Materials and Methods

### 

#### Samples

We used DNA from 24 western chimpanzees *(Pan troglodytes verus)* that were wild caught or known to be unrelated based on recent pedigrees. Twelve samples (Annaclara, Frits, Hilko, Liesbeth, Louise, Marco, Oscar, Regina, Socrates, Sonja, Yoran, and Yvonne) are from the collection stored at the Max Planck Institute for Evolutionary Anthropology, Leipzig, Germany, while 12 other samples (NDH0311G1, NDH0312G1, NDH0313G1, NDH0314G1, NDH0317G1, NDH0320G1, NDH0321G1, NDH0322G1, NDH0325G1, NDH0326G1, NDH0328G1, and NDH0329G1) were kindly provided by P. Morin and the Primate Foundation of Arizona.

#### Primer design

We amplified 9,491 bp from the TAP2 region, corresponding to base pairs 113102–122585 of the sequence from Beck et al. (1996) (see Supporting Information); the slight discrepancy in the number of base pairs is due to indels. To minimize the chance of allelic dropout, we designed the PCR primers such that most of the sequence would be amplified by two independent sets of primers. The 20 overlapping primer sequences are listed in Protocol S1.

#### PCR and DNA sequencing

DNA amplification reactions contained 250 μM of each dNTP, 1–2 mM MgCl_2_, PCR buffer (10 mM Tris-HCl, 50 mM KCl; pH 8.3), 0.5 U of Taq DNA polymerase (all reagents from Roche, Basel, Switzerland), and 10 pmol of each primer. We used 50–100 ng of DNA in each 30 μl PCR. Amplification conditions for all regions were the following: incubation for 3 min at 94 °C, 35 cycles (45 s at 94 °C, 1 min at 45–62 °C, and 1 min at 72 °C) and a final elongation of 5 min at 72 °C. A nested PCR was performed to obtain regions 6 and 7 by using the product of the primers Tap2 5 5′ and Tap2 8 3′ as a template. PCR products were separated from primers and unincorporated dNTPs by treatment with a solution of 10% PEG 8000/1.25 M NaCl followed by centrifugation. PCR products were then air dried and resuspended in 10–15 μl of H_2_O.

Sequencing reactions consisted of 1 μl of ABI Prism BigDyeTM Terminators version 2.0 (Perkin Elmer Biosystems, Torrance, California, United States), 8–10 ng of purified PCR product, and 1 μl of 2.5 μM primer (the same primers used for PCR) in a volume of 7 μl. Cycling conditions were 96 °C for 2 min and then 35 cycles of 96 °C (20 s), annealing temperature (30 s), and 60 °C (4 min). Isopropanol-precipitated cycle sequencing products were run on an ABI 3730 DNA analyzer. Base calling was done with ABI Prism DNA Sequencing Analysis version 5.0 and ABI Basecaller. BioEdit version 5.0.6 was used for sequence analysis and alignment.

In total, 2-fold coverage of a 9,370 bp sequence was obtained for each individual; these are available from GenBank (see Supporting Information). Most of the region was sequenced from both DNA strands. However, due to the presence of insertions, deletions, and T or A stretches, this was not possible for a subset of segments; for these, 2-fold coverage was achieved by sequencing the same strand. For segment 6, we did not obtain reliable sequence data for all individuals (due to suspected allelic dropout); we therefore excluded this region of 487 bp. Otherwise, there are no missing data. SNPs were identified by visualization of the chromatograms using BioEdit version 5.0.6. The polymorphism data used for the analyses are available in Protocol S1.

#### Data analysis

We estimated the population mutation rate, *θ* = 4*N*_e_ *μ* (*N*_e_ is the diploid effective population size and *μ* is the mutation rate per generation), using Watterson's estimator, *θ*_W_ (Watterson 1975), based on the number of segregating sites in the sample. We also calculated a commonly used summary of the allele frequency spectrum, Tajima's *D* (Tajima 1989); both *D* and *θ*_W_ were calculated with DNAsp (Rozas and Rozas 1999). We used the *D* statistic to test the fit of the standard neutral model (of a random mating population of constant size) to allele frequencies in western chimpanzees. Specifically, we ran 10^4^ coalescent simulations of the standard neutral model with the same number of chromosomes and base pairs as in the actual data, with *θ* equal to *θ*_W,_ and with the population recombination rate equal to the estimated value (see below). We then tabulated the proportion of simulated runs with a Tajima's *D* value as or more extreme than that observed.

We calculated the GC content of the region and searched for sequence motifs previously associated with recombination hotspots (Badge et al. 2000; Petes 2001; Wall et al. 2003) using the program “scan_for_matches” available from http://bioweb.pasteur.fr/seqanal/interfaces/scan_for_matches.html. The list of motifs found in the human and chimpanzee sequences is given in Protocol S1.

#### Analyses of LD

To assess the support in the polymorphism data for a recombination hotspot, we used the Product of Approximate Conditionals (PAC) model of Li and Stephens (2003). Assuming haplotypes are known, the method considers each one in turn and attempts to represent it as a mosaic of the previously considered haplotypes. Qualitatively, the larger the regions over which haplotypes tend to resemble one another, the fewer the pieces required in each mosaic, and the lower the estimates of the recombination rates. The method uses simplistic assumptions about population demography to quantify this qualitative relationship and hence to estimate recombination rates across the region.

More formally, the model of Li and Stephens (2003) defines the probability of observing haplotypes *H* given the underlying recombination parameters *α* (which in our case may include the background recombination rate and the hotspot location and intensity; see below). This can be used directly to estimate *α* from *H* in situations where haplotypes have been experimentally determined (e.g., Li and Stephens 2003). However, in our case the chimpanzee haplotypes are not known. Rather, we have genotype data *G* and we wish to estimate *α* from *G.* A simple approximate solution to this would be first to use a statistical method (e.g., that of Stephens et al. 2001) to obtain an estimate H^ for the haplotypes *H* from the genotypes *G*, and then to estimate *α* from H^. However, a risk of this approach is that overconfident conclusions will be drawn by ignoring uncertainty in the estimated haplotypes. A better solution, and the approach we take here, is to jointly estimate *H* and *α* from *G*, or, more specifically, to obtain a sample from the joint posterior distribution, Pr(*H*, *α* | *G*). To do so, we start with an initial guess for the haplotypes, and iterate the following steps: (i) estimate a new value for *α*, using the current estimate of *H* and (ii) estimate a new value for *H*, using the genotypes *G* and the current value for *α*. Step (i) is performed using the PAC-B model of Li and Stephens (2003) and the priors on *α* described below. Step (ii) is performed by using the method for haplotype inference described in Stephens and Donnelly (2003), but replacing the conditional distribution that they use (which ignores recombination) with the conditional distribution of Fearnhead and Donnelly (2001) (which takes into account recombination) computed using two quadrature points. (Actually, we modified the Fearnhead and Donnelly conditional distribution slightly, replacing the equation *q_i_ = z_i_ρ/(j+ z_i_ρ)* in their Appendix A with *q_i_ =1−exp(−z_i_ρ/j)*.) Both the PAC-B model and the Fearnhead and Donnelly conditional require the specification of a mutation parameter, *θ*, and a mutation process. In each case, we used the value of *θ* given in Li and Stephens (2003) and a mutation process whereby each mutation event at a biallelic site results in a change from one allele to the other.

This iterative scheme defines a Markov chain whose stationary distribution is the distribution Pr(*H*, *α* | *G*) from which we wish to sample. Provided that the algorithm is run for sufficiently long, the estimates of *α* obtained each iteration provide a sample from the distribution Pr(*α* | *G*), and thus allow *α* (i.e., the underlying recombination process) to be estimated directly from *G,* taking full account of the fact that the actual underlying haplotypes are not known. The algorithm is implemented within the software package PHASE version 2.1, which is available online at http://www.stat.washington.edu/stephens/software.html.

We considered two versions of the simple hotspot model of Li and Stephens (2003). In this model, there is a single hotspot of constant intensity *λ*. Crossovers occur as a Poisson process (i.e., there is no interference) of constant rate *r* (per base pair) outside the hotspot and of constant rate *λr* inside the hotspot; gene conversion is not explicitly modeled. In the first version, we assumed that, if present, the hotspot is at the same location as estimated by sperm typing in humans (4180–5417). (This location is not precisely the same as the one used by Li and Stephens [2003], which is why our estimates differ from theirs.) There are two parameters to be estimated: the background population recombination parameter *ρ* (= 4*N*_e_ *r*, where *N*_e_ is the effective population size) and *λ*. We assumed a priori that a hotspot exists with probability 0.5 and that, if the hotspot exists, *λ* is between one and 100. Specifically, we assumed that *λ* = 1 with probability 0.5 and otherwise that log_10_ *(λ)* is uniformly distributed on (0, 2). The prior on *ρ* is uniform on log_10_ *(ρ)* in the range (−8, 3), which covers all plausible values.

In the second version, we assumed that the location and width of the hotspot are unknown and to be estimated along with *λ* and *ρ*. In this case, we assumed a priori that the hotspot exists with probability 0.18 (corresponding to an assumption that a hotspot occurs roughly once per 50 kb of sequence), that the center of the hotspot is equally likely to be anywhere along the length of the sequence, and that the width of the hotspot is between 200 and ∼4,000 bp (specifically, we assumed that the width had a normal distribution, with a mean of 0 bp and a standard deviation of 2,000 bp, truncated to lie above 200 bp). Priors on *ρ* and on *λ* (conditional on there being a hotspot) are as in the first version.

To allow for potential problems with convergence of this Markov chain Monte Carlo algorithm, we ran the algorithm ten times for each analysis, using different seeds for the pseudorandom number generator. For each run, we obtained a point estimate of the parameters (using sample posterior medians) and posterior probabilities. The reported estimates are the median of the estimates obtained from the ten runs.

To test how likely we would be to obtain such weak support for a hotspot in the LD data if there were in fact a hotspot similar to the one in humans, we ran 200 coalescent simulations of the standard neutral model (Hudson 1990) with the same number of base pairs and sample size as the actual data (48 chromosomes), a hotspot of intensity *λ* = 11, and *θ* = *θ*_W_. Haplotypes were randomly paired to form genotypes and phase information was ignored. The data were masked to mimic the actual data structure, i.e., they included a gap of 487 bp in the same position. We then counted the proportion of simulated data sets for which our estimate of the posterior probability was as low as observed or lower (using the first version of the Li and Stephens [2003] model). Since we obtained estimates for the simulated data in the same way as for the actual data, significance values obtained from this analysis are valid independent of the convergence, or even the correctness, of the Markov chain Monte Carlo scheme. In the first set of 200 simulations, we used *ρ* = ρ^*,* the background rate that we estimated from the western chimpanzee data. In the second set of simulations, we used *ρ* = 4N^_e_r^ = 2.7 × 10^−4^ per bp, where N^_e_ = 17,100 is an estimate of the effective population size of western chimpanzees (based on Fischer et al. 2004) and r^ = 0.4 cM/Mb is the rough estimate of the background recombination rate reported in Jeffreys et al. (2000).

## Supporting Information

Protocol S1 Supplementary Materials (91 KB DOC).

### Accession Numbers

The GenBank (http://www.ncbi.nlm.nih.gov/) accession number for the human TAP2 region of Beck et al. (1996) is X87344. The numbers for the 9,370-bp sequences obtained from the 24 western chimpanzees are AY559252–AY559299.

## Abbreviations

LD: linkage disequilibrium
PAC model: Product of Approximate Conditionals model
SNP: single nucleotide polymorphism.
