# Supplementary material for: Absence of the TAP2 Human Recombination Hotspot in Chimpanzees
Source: PLoS Biol. 2004 Jun 15;2(6):e155. doi: 10.1371/journal.pbio.0020155 (PMC423135; doi:10.1371/journal.pbio.0020155)
Supplement: Protocol S1 — (91 KB DOC). [file pbio.0020155.sd001.doc]

**Supplementary materials**.

I. Primers used in the study

All are listed 5’ to 3’:

TAP2-1-5’ GAGAATCACTTGAACCTGGGAG

TAP2-1-3’ AAGGAAACCCCAGGAAGAAATA

TAP2-2-5’ TTGTCCACAGTGTACCACATGA

TAP2-2-3’ ATCTTGCAATTCAGCATGACAC

TAP2-3-5’ TATTTCTTCCTGGGGTTTCCTT

TAP2-3-3’ ACCACCTCCCAAACTAAAAACA

TAP2-4-5’ CATGATGTGTCATGCTGAATTG

TAP2-4-3’ CGGGCTCTCCTAAATAGAAGGT

TAP2-5-5’ ATAGAACAAGAACCAAAGCCCA

TAP2-5-3’ GGCGGTTACTCTGGGATATAAA

TAP2-6-5’ GGACAACAGATAAAGTTGCCCT

TAP2-6-3’ CTGTCAACGGATACGAGATGAG

TAP2-7-5’ AAGTGACTACCCACTCCACGCT

TAP2-7-3’ AGTAAAGCCGCGTCCACCAGC

TAP2-8-5’ CATCTCGTATCCGTTGACAGAG

TAP2-8-3’ AGGGCCTAGAAATGGAGTTAGG

TAP2-9-5’ CCTCACTATTCTGGTCGTGTGA

TAP2-9-3’ TGAAATGGTTTTCGAGGGTACT

TAP2-10-5’ GGTGAATGTAGTCACCATTCTG

TAP2-10-3’ ATGACTGCACCTAACACTGTGG

TAP2-11-5’ GATGGTGGACCCACCAGATTAT

TAP2-11-3’ GAGGGGCAAAAGAGAAAGAAAT

TAP2-12-5’ GGCAGGGAATTTATTATTGCAG

TAP2-12-3’ CCTCTGAACATCCTCCTTCACT

TAP2-13-5’ CATCTCCCTCCCCTCTTATTCT

TAP2-13-3’ TATACCTTCTCCCCTAACGGCT

TAP2-14-5’ CGGATACCACCCTGATGAGTAA

TAP2-14-3’ GGTTGGGGGTAGTAGGCAGTTA

TAP2-15-5’ ATAGGAAGGGGAGATGATAGGC

TAP2-15-3’ TCTTTTGTAAATTGCCCGATCT

TAP2-16-5’ CTCCGTTGTACCTTCTGTCTCC

TAP2-16-3’ CAGAAAGTGGTGTTGATGAGGA

TAP2-17-5’ AGATCGGGCAATTTACAAAAGA

TAP2-17-3’ CTTGGATATAACACCAAACGCA

TAP2-18-5’ TCTTTGCCCATTTTCTGATTTT

TAP2-18-3’ TGTTCAAGGGGCTCTTTATAGC

TAP2-19-5’ CCTTCCTTCCTGATTCAGACAC

TAP2-19-3’ GAAGGCCACAAAGAAAAAGAGA

TAP2-20-5’ GCTATAAAGAGGCCCTTGAACA

TAP2-20-3’ CTCATACACATACACTACATGG

II. Polymorphism data used for analyses.

Labels refer to the western chimpanzees listed in Methods. The genotypes are given as pseudohaplotypes, *i.e*. the phase is unknown. Positions are given with respect to the sequence from Jeffre*ys et a*l. (2000), available at <http://www.le.ac.uk/ge/ajj/tap2/SEQdata.html/>.

Positions:

536 552 556 557 568 703 1114 1211 1613 1808 2090 2176 2417 2669 3271 3319 3491 3600 3804 3841 4006 4062 4075 4196 4274 4415 4451 4452 4466 4572 4613 4887 4927 5197 5503 5569 5775 5789 5854 5868 6342 6388 6796 7429 7978 8089 8113 8377 8621 8824 8974 9141 9159 9501 9649 9680 9759

> 311

A T A C C C T G G A G G C A A G A A T C T T C C G A T A G A C G C C A G T C C C T A G T T G T C C C C G A C C T C

A T A C C C T G G A G G C A A A A A T C T T C T G A T A G A C G C C A A T C C C T A G T T G T C C T C G A C A T C

> 312

G C A C C C T G G A G G C A A G A G T C T T C C G A T A G A C G C C A G T C C C T A G T T G C C C C C G A C C T C

A T G T C T T G A A A A T A A A G A T C T T T C G A T A G A C C T C A G T C C C T A G T T G T A C C C G A T A T C

> 313

G C A C C C T G G A G G C A A G A G T C T T C C G A T A G A C G C C A G T C C C C A G T T G T C C C C G G C C T C

A T G T C T T G A A A A T A A A A A T C T T C C G A T A G A C C C C A G T C C C T A A T T G T C C C C A A C A A T

> 314

G C A C C C T G G A G G C A A G A G T C T T C C G A T A G A C C C C A G T C C C C A G T T G C C C C C G G C C T C

A T G T C T T G A A A A T A A A A A T C T T C C G A T A G A C C C C A G T C C C T A A T T G T A C C T A A C C A T

> 317

G C A C C C T G G A G G T G G G G G C C C C C C A G G A G G C C C C A G C C C C C G G T C G T C C C C G A C C A C

A T G T T T T G A A A A T A A G A A T T T T C C A A T A T A T C C T A G T G T T C A A T T C T C T C C A A C C A T

> 320

G C A C C C T G G A G A T A A G G G C C C C C C A G G A G G C C C C A G C C C C C G G T C G T C C C C G G C C A C

A T G T T T T G A A A A T G A G A A T C T T C C A A T A T A T C C T A G T G T T C A A T T C T C T C C A A C C A T

> 321

A T A C C C T G G A G G C A A G A A T C T T C C G A T A G A C G C C G G T C C C T A G T T G C C C C C G A C C T C

A T A C C C T A G A G G C A A A A A T C T T C C G A T A G A C G C C A G T C C C T A G C T G T C C C C G A C A T C

> 322

G C A C C C T G G A G G C A A G G G T C T T C C G A T A G A C G C C G G T C C C T A G T T G C C C C C G A C C T C

A T G T C T T G A A A A T A A A A A T C T T C C G A T A G A C C C C A G T C C C T A G C T G T C C C C G A C A T C

> 325

A T A C C C T G G A G G C A A G G A T C T T C C G A T A G A C G C C A G T C C C T A G T T G T C C C C G G C C T C

A T A C C C T A G A G G C A A A A A T C T T C T G A T A G A C G C C A G T C C C T A G T T G T C C T C A A C A A T

> 326

G C A C C C T G G G G G C A A G A G T C T T C C G A T A G A C G C C A G T C C C C A G T T G T C C C C G G C C T C

A T G T C T T A A A A A T A A A G A T C T T C C A A T A G A C C C C A G T C C C T A A T T G T A C C C A A C A A T

> 328

A T A C C C T G G A G G C G A G G A C C C C C C G G G A G G C G C C A G C C C C C G G T T G T C C C C G A C C T C

A T A C T C T A G A G A T A A A A A T C T T T C A A T A T A T C T T A G T G T T T A G T T C T A T C C G A C C A C

> 329

A T A C T C C G G A G G C A A G G A C C C C C C G G G A G G C G C C A G C C C C C G G T C G C C C C C G A C C T C

A T A C T C T G G A G A T A A A A A T C T T C C A A T A T A T C C T A G T G T T T A G T T C T A T C T G A C C A C

> Annaclara

G C A C C C T G G A G G C A A G A G T C T T C C G A T A G A C G C C A G T C C C C A G T T G T C C C C G G C C T C

A T G T C T T A A A A A T A A A A A T C T T C C A A T A G A C C C C A G T C C C T A A T T G T C C C C A A C A A T

> Frits

A T A C C C T G G A G G C A A A A A T C T T C C G A T C G A C G C C A G T C C C T A G T T G T C C C C G A C A T C

A T A C C C T A G A G G C A A A A A T C T T C T G A T A G A C G C C A G T C C C T A G T T G T C C T C G A C A T C

> Hilko

G C G T C T T G A A A A C A G G A G T C T T C C G A T A G A C C C C A G T C C C C A G T T G T C C C C G G C C T C

G C G T C T T G A A A A C A A G A G T C T T C C A A T A G A C C C C A G T C C C T A A T T G T C C C C A A T A A T

> Liesbetha

A T A C C C T G G A G G C A A A A A T C T T C T G A T A G A C G C C A G T C C C T A G T T G T C C T C G A C A T C

A T A C C C T G G A G G C A A A A A T C T T C T G A T A G A C G C C A G T C C C T A G T T G T C C T C G A C A T C

> Louise

A T A C C C T G G A G G C A A A A A T C T T C C G A T A G A C G C C G G T C C C T A G T T G T C C C C G A C A T C

A T A C C C T A G A G G C A A A A A T C T T C C G A T A G A C G C C A G T C C C T A G C T G T C C T C G A C A T C

> Marco

A T A C C C T G G A G G C A A A A A T C T T C C G A T C G A C G C C A G T C C C T A G T T G T C C C C G A C C T C

A T A C C C T G G A G G C A A A A A T C T T T T G A T A G A C G T C A G T C C C T A G T T G T A C T C G A C A T C

> Oscar

A T A C C C T G G A G G C A A G G A C C C C C C G G G A G G C G C C A G C C C C C G G T C G T C C C C G A C C T C

A T A C T C T G G A G A T G A A A A T C T T C T A A T A T A T C C T A G T G T T T A G T T C T C T T C G A C A A C

> Regina

A T A C C C T G G A G G C G A G G A C C C C C C G G G A G G C G C C A G C C C C C G G T C G T C C C C G A C C T C

A T A C T C T A G A G A T A A G A A T C T T C C A A T A T A T C C T A G T G T T T A G T T C T C T C C G A C A A C

> Socrates

G C A C C C T G G A G G C A A G A G T C T T C C G A T A G A C G C C A G T C C C T A G T T G T C C C C G A C A T C

A T G T C T T A A A A A T A A A A A T C T T C C G A T A G A C C C C A G T C C C T A G T T G T C C C C G A T A T C

> Sonja

A T A C C C T G G A G G C A A A A A T C T T C C G A T A G A C G C C G G T C C C T A G T T G T C C C C G A C C T C

A T A C C C T A G A G G C A A A A A T C T T C C G A T A G A C G C C A G T C C C T A G C T G T C C C C G A C A T C

> Yoran

G T A C C C T G G A G G C A A G A G T C T T C C G A T A G A C G C C G G T C C C C A G T T G T C C C C G G C C T C

A C G T C T T G A A A A T A A A A A T C T T C C A A T A G A C C C C A G T C C C T A A C T G T C C C C A A C C A T

> Yvonne

G C A C C C T G G A A G T A A G G G C C C C C C A G G A G A C C C C A G C C C C C G G T C G T C C C C G G C C A C

A T G T T T T G A A A A T G A G A A T C T T C C A A T A T G T C C T A G T G T T C A A T T C T C T C C A A C C A T

III. Search for sequence motifs previously associated with recombination hotspots.

Unless indicated otherwise, all searches used the interface available from <http://bioweb.pasteur.fr/seqanal/interfaces/scan_for_matches.html> and were run with the following tolerance levels: 0 for a mismatch, 0 for an indel.

The locations of the motifs are indicated by []. Positions are given with respect to the sequence from Jeffre*ys et a*l. (2000), available at <http://www.le.ac.uk/ge/ajj/tap2/SEQdata.html/>.

In the consensus sequences, the following equivalencies are used:

R = (A,G)

Y = (T,C)

W = (A,T)

S = (G,C)

M = (A,C)

K = (G,T)

H = (A,T,C)

B = (G,C,T)

V = (G,A,C)

D = (G,A,T)

N = (A,G,C,T)

1) Search for motifs listed in Smi*th et a*l. (1998).

Possible location where Pur element promotes duplex opening: ATATATTTT

>humantap2:[5330,5338]

>chimptap2:[5330,5338]

(ATTTT)_n_

none

WAWTTDDWWWDHWGWHMAWTT

none

2) Search for additional motifs listed in Bad*ge et a*l. (2000).

CHI: GCTGGTGG

>humantap2:[3527,3534]

>humantap2:[3794,3801]

>chimptap2:[3527,3534]

>chimptap2:[3794,3801]

ade6-M26 heptamer: ATGACGT

none

LTR-IS: TGGAAATCCCC

none

retroransposon LTR: TCATACACCACGCAGGGGTAGAGGACT

none

XY32 homopurine-pyrimidine H-palindrome: AAGGGAGAARGGGTATAGGGRAAGAGGGAA

none

human minisatellite core sequence: GGGCAGGARG

GGGCAGGAAG:

>humantap2:[5547,5556]

>chimptap2:[5547,5556]

human hypervariable minisatellite sequences: GGAGGTGGGCAGGARG,AGAGGTGGGCAGGTGG

none

pur: GGNNGAGGGAGARRRR

>humantap2:[8784,8799]

GGAAGAGGGAGAGAAA

translin: GCNC[A/T][G/C][G/C][A/T] N(0-2) GCCC[A/T][G/C][G/C][A/T] -> GCNCWSSW N(0-2) GCCCWSSW

GCNCWSSWGCCCWSSW

GCNCWSSWNGCCCWSSW

GCNCWSSWNNGCCCWSSW

none

human replication origin consensus: WAWTTDDWWWDHWGWHMAWTT

none

S.cerevisiae ARS: WTTTATRTTTW

none

S.pombe ARS: WRTTTATTTAW

none

scaffold attachment regions:

AATAAAYAAA: none

TWWTDTTWWW or TTWTWTTWTT: TTTTTTTTTT

>humantap2:[2301,2310]

>humantap2:[3201,3246]

>humantap2:[3247,3256]

>chimptap2:[3201,3246]

>chimptap2:[8105,8114]

>chimptap2:[8278,8287]

WADAWAYAWW:

AAGAAACATT

>humantap2:[2749,2758]

>chimptap2:[2749,2758]

TAGAAACATA

>humantap2:[5323,5332]

>chimptap2:[5323,5332]

AAAATACAAA

>humantap2:[9736,9745]

>chimptap2:[9736,9745]

topoisomerase II binding site: GTNWAYATTNATNNR

none

3) Search for additional motifs listed in Petes (2001).

β hotspot:

(CCGNN)_12_:

none

(CGGATCCG)_4_:

none

GC content: human 46.8%, chimp 47.0%

(GT)_n_:

none (no more than 3 repeats)

CoHR: -AG-T-GAACAA-ATAATC-AAAAAAAAAAAACTGT--C----C-A------

Cf. Blumental-Per*ry et a*l. (2000)

none

4) Search for additional motifs listed in Wa*ll et a*l. (2003).

(RY)_n_:

none (no more than 6 repeats)

WEB-THERMODYN: <http://wings.buffalo.edu/gsa/dna/dk/>

no difference

Helical stability of the DNA sequence.

References:

Badge RM, Yardley J, Jeffreys AJ, Armour JA (2000) Crossover breakpoint mapping identifies a subtelomeric hotspot for male meiotic recombination. Hum Mol Genet 9:1239-1244

Blumental-Perry A, Zenvirth D, Klein S, Onn I, Simchen G (2000) DNA motif associated with meiotic double-strand break regions in *Saccharomyces cerevisiae*. EMBO Rep 1:232-238

Jeffreys AJ, Ritchie A, Neumann R (2000) High resolution analysis of haplotype diversity and meiotic crossover in the human TAP2 recombination hotspot. Hum Mol Genet 9:725-733

Petes TD (2001) Meiotic recombination hot spots and cold spots. Nat Rev Genet 2:360-369

Smith RA, Ho PJ, Clegg JB, Kidd JR, Thein SL (1998) Recombination breakpoints in the human beta-globin gene cluster. Blood 92:4415-4421

Wall JD, Frisse LA, Hudson RR, Rienzo AD (2003) Comparative linkage disequilibrium analysis of the ß-globin hotspot in primates. Am J Hum Genet 73:1330-1340.
